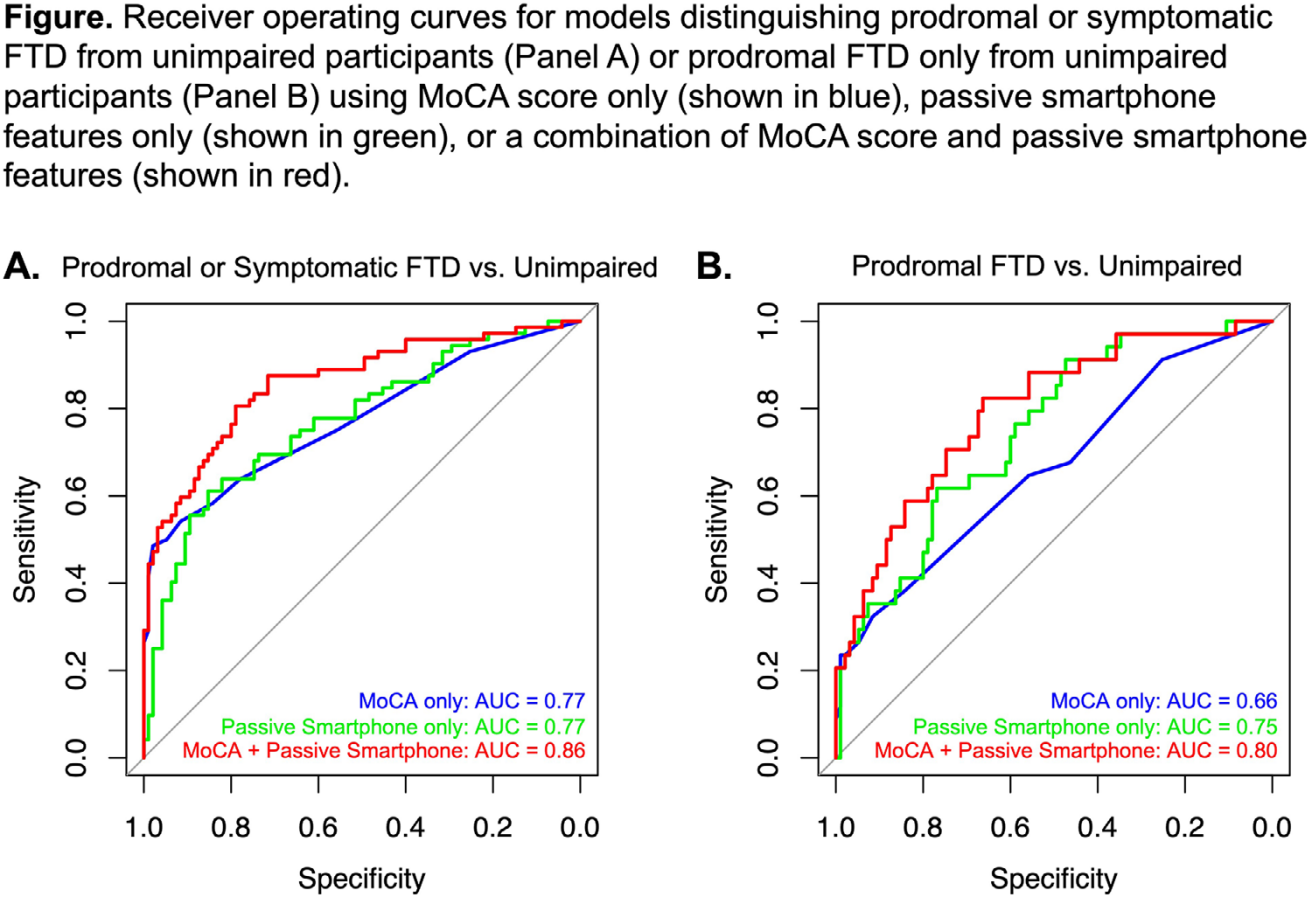# Passively collected data from smartphones improves detection of frontotemporal dementia compared to the MoCA

**DOI:** 10.1002/alz.090571

**Published:** 2025-01-03

**Authors:** Emily W. Paolillo, Kaitlin B. Casaletto, Jack C. Taylor, Hilary W. Heuer, Amy B. Wise, Sreya Dhanam, Mark E. Sanderson‐Cimino, Brandon R. Palacios, J. Clayton Young, Mai Anh Bui, Rowan Saloner, Shubir Dutt, Anna M. VandeBunte, Claire J. Cadwallader, Joel H. Kramer, Walter K. Kremers, Leah K. Forsberg, Brad F. Boeve, Howard J. Rosen, Adam L. Boxer, Adam M. Staffaroni, the ALLFTD Consortium

**Affiliations:** ^1^ Memory and Aging Center, UCSF Weill Institute for Neurosciences, University of California, San Francisco, San Francisco, CA USA; ^2^ Memory and Aging Center, UCSF Weill Institute for Neurosciences, San Francisco, CA USA; ^3^ Mayo Clinic, Rochester, MN USA; ^4^ Department of Neurology, Mayo Clinic, Rochester, MN USA; ^5^ Memory and Aging Center, Weill Institute for Neurosciences, University of California, San Francisco, San Francisco, CA USA

## Abstract

**Background:**

Frontotemporal dementia (FTD) is a common young‐onset dementia. Challenges to in‐person FTD evaluations (e.g., behavioral symptoms, disease rarity), highlight the need to develop remote, low‐burden assessment techniques. A growing literature supports passive digital phenotyping for monitoring neurobehavioral change. Thus, we examined the utility of passively collected data from smartphones to detect prodromal or symptomatic FTD compared to that of the Montreal Cognitive Assessment (MoCA), a common cognitive screener.

**Method:**

199 adults enrolled in the ALLFTD Mobile App study (mean age = 53.4 [SD = 15.2]; 58% women) completed up to 6 months of passive smartphone monitoring via the ALLFTD Mobile App. 55% were unimpaired [CDR®+NACC FTLD = 0], 22% had prodromal FTD [CDR®+NACC FTLD = 0.5], and 23% had symptomatic FTD [CDR®+NACC FTLD≥1] with a variety of syndromic presentations. Battery percentage was collected frequently (median interval = 45 min), and total daily battery usage was calculated as a proxy for smartphone use (higher battery usage = more smartphone use). Daily step counts were also passively collected. Logistic regression classified prodromal or symptomatic FTD vs. unimpaired as a function of: MoCA score [Model 1]; 7 passive features characterizing average, inter‐day variability, and changes over time in smartphone use and movement [Model 2]; or passive smartphone features and MoCA [Model 3]. Analyses were repeated excluding participants with symptomatic FTD to detect prodromal FTD vs. unimpaired.

**Result:**

The area under the curves (AUCs) for detecting prodromal or symptomatic FTD (vs. unimpaired) from the MoCA alone [Model 1] and passive smartphone features alone [Model 2] were 0.77 (95%CI: 0.70‐0.85) and 0.77 (95%CI: 0.70‐0.84), respectively. Their combination [Model 3] resulted in a significantly improved AUC of 0.86 (95%CI: 0.80‐0.92) compared to Model 1 (*p* = 0.001). Significant Model 3 predictors included average daily battery usage (*p* = 0.02), slopes of change in step count (*p* = 0.04), and MoCA score (*p*<0.01). When distinguishing prodromal FTD from unimpaired, passive smartphone features alone (AUC = 0.75) were significantly better than the MoCA alone (AUC = 0.66; *p*<0.01), and their combination had the largest AUC (0.80).

**Conclusion:**

Results support the utility of novel passively collected information from smartphones for detecting and monitoring FTD without contributing to assessment burden. With continued validation, passive digital monitoring methodologies have potential to increase access to dementia care.